# Geosensors to Support Crop Production: Current Applications and User Requirements

**DOI:** 10.3390/s110706656

**Published:** 2011-06-27

**Authors:** Sirpa Thessler, Lammert Kooistra, Frederick Teye, Hanna Huitu, Arnold K. Bregt

**Affiliations:** 1 Plant Production Research, MTT Agrifood Research Finland, Latokartanonkaari 10, 00790 Helsinki, Finland; E-Mails: frederick.teye@mtt.fi (F.T.); hanna.huitu@mtt.fi (H.H.); 2 Laboratory of Geo-Information Science and Remote Sensing, Wageningen University, P.O. Box 47, 6700 AA Wageningen, The Netherlands; E-Mails: lammert.kooistra@wur.nl (L.K.); arnold.bregt@wur.nl (A.K.B.)

**Keywords:** sensors, sensor networks, crop production, agriculture

## Abstract

Sensor technology, which benefits from high temporal measuring resolution, real-time data transfer and high spatial resolution of sensor data that shows in-field variations, has the potential to provide added value for crop production. The present paper explores how sensors and sensor networks have been utilised in the crop production process and what their added-value and the main bottlenecks are from the perspective of users. The focus is on sensor based applications and on requirements that users pose for them. Literature and two use cases were reviewed and applications were classified according to the crop production process: sensing of growth conditions, fertilising, irrigation, plant protection, harvesting and fleet control. The potential of sensor technology was widely acknowledged along the crop production chain. Users of the sensors require easy-to-use and reliable applications that are actionable in crop production at reasonable costs. The challenges are to develop sensor technology, data interoperability and management tools as well as data and measurement services in a way that requirements can be met, and potential benefits and added value can be realized in the farms in terms of higher yields, improved quality of yields, decreased input costs and production risks, and less work time and load.

## 1. Introduction

Agricultural production is vital for feeding the human population. Production of crops, however, show variation in space and time due to changes in weather conditions, different management practices and other external factors. Information on the day-to-day factors influencing crop growth has been important for farmers for ages. In the past farmers mainly used direct human observations to recognize these factors. In the last decades, however, more and more automatic sensor systems such as soil moisture sensors, weather stations and satellite or airborne sensors have been adopted [1]. Automatic sensors and sensor networks enable local and (near) real-time observations and monitoring, and may foster more sustainable crop production practices and, thus, lower negative environmental impacts of agriculture and food safety risks.

Sensor and communication technology has quickly developed from off-line sensors using field loggers and manual downloading to wireless on-line sensor networks, and is moving toward interoperable and autonomous sensor webs [2]. This sensor web concept is based on the Sensor Web Enablement (SWE) framework of the Open Geospatial Consortium (OGC). Within this framework, standard protocols, interfaces and web services to discover, task, exchange and process data from different sensors and sensor networks have been defined [3–5]. Geo-sensors were loosely defined by Nittel *et al*. in 2008 [6] as sensors that monitor phenomenon in a geographical place. Until now the development of sensors and sensor networks has mainly been driven by technological innovations. However, this technological push has been supported by growing needs for food and fiber, demands for reducing environmental effects of agriculture and concerns over food safety [7]. Also increasing competition in the global food market and decreasing profitability of European farms have been seen to support adoption of new technology in the farms [7,8].

Agriculture benefits from high temporal measuring resolution, real-time data transfer and high spatial resolution of sensor data that shows in-field variations [2,9–12]. Sensor technology has the potential to provide added value for agriculture e.g., for improving yield quality or for decreasing costs or risks in production. Currently sensor technology has been most commonly applied in real-time weather monitoring for support of management practices, and in precision agriculture [10,13]. Maturing technology has also enabled first commercial and operational applications in agriculture [14].

Sensor networks in agricultural applications have been studied with growing interest. Past papers mainly focused on technical issues (hardware, software, energy-consumption and communication) [4,9,11,13,14,16–18]. Also recent review articles [13,15,18] on sensor technology and sensor networks in agriculture had a predominantly technical perspective. More and more queries have been made for studies that focus on applications and operational use of sensor technology in the field of agriculture and environmental monitoring [14]. As far as we know, agricultural applications of sensor networks have not been analyzed or reviewed from a practical application and user point of view. Relevant questions when sensor networks are implemented in operational crop production and applications are: How do they support crop production? What added-value do they bring to production process? How do users benefit? What are the risks and bottlenecks involved?

In the present paper we aim to explore these questions in crop production process. The analysis is done by comprehensive literature review and the analysis of two sensor network use cases. Literature review of geo-sensor network applications is limited to crop production including *in-situ* or portable sensors and sensors mounted in field or airborne vehicles or machines.

## 2. Sensors and Sensor Network Applications in Arable Crop Production

### 2.1. Literature Review

#### 2.1.1. Sensing of Growing Conditions

Many of farming decisions are based, at least partly, on weather information. Because weather is an important factor in agriculture, many agro-meteorological networks were founded decades ago to support agriculture [2]. Most countries provide agro-meteorological services by integrating national weather data with soil and agricultural data; such as crop growth models, plant disease and pest information. These are used for timing tasks like plant protection and irrigation. Many countries also offer alerting or forecast services and maintain separate agrometeorological station networks [19]. The first networks were based on meteorological stations and voluntary observers, but already in the 80s automated weather stations started to be commonly available [20,21]. These networks, some of them still operational, offered weather and soil information combined with agricultural models to support farmers’ management decisions [20,22].

The regional agro-meteorological sensor network, AgWeatherNet, from Washington State (USA) [10] is a recent example of a commercially successful sensor network application. It integrates biological knowledge and sensor data to provide decision support for fruit tree farmers. Also applications for irrigation system performance, crop-load monitoring and manure spreading employ weather data acquired by AgWeatherNet.

Knowledge of the spatial variability of soil attributes within an agricultural field is critical for successful site-specific crop management. At the start of the growing season, the farmer is interested in the actual soil status (e.g., compaction, nutrient balance, pre crop) to decide on the required soil preparation activities. During the growing season, information on nutrient and pesticide fluxes is relevant for proper timing of management decisions. However, manual soil sampling and laboratory testing is tedious and expensive. Several studies have presented alternative soil sensing techniques which are able to assess this variability. Two main types of soil sensing techniques can be distinguished: (1) proximal sensors mounted on vehicles for on-the-go measurements [23], and (2) wireless sensor networks with individual nodes located at different positions and depths within an agricultural parcel [24].

Proximal sensing techniques can be defined as field based techniques that can be used to measure soil chemical, physical, biological and mineralogical properties from a distance of approximately less than 2 m above the soil surface [25]. A broad variety of on-the-go proximal sensing techniques has been applied ranging from diffuse reflectance spectroscopy using Visible-Near-Infrared (VIS-NIR) [26,27] or Mid-Infrared (MIR) wavelengths [27], Gamma-Ray Radiometry (GRR) [28], Ground Penetrating Radar (GPR) [29], and Electrical Conductivity (EC) [30]. VIS-NIR and MIR can provide information of the top-soil on several soil properties (e.g., soil organic carbon, texture, nitrogen content, pH) [15]. Viscarra Rossel *et al*. [27] compared the performance of different wavelength ranges using VIS, NIR and MIR. Prediction accuracy varied greatly depending on the soil property. GRR and EC provided subsurface information and for example the derived textural information could be adopted as a-priory variable to improve predictions based on VIS-NIR spectroscopy [31]. GPR also quantifies soil properties of the subsurface and especially is capable to provide information about soil moisture. Integration of different sensing techniques by selecting a complementary set of sensors, may improve the estimation of soil properties [32]. These so-called multi-sensor platforms [24] would allow increased confidence as independent measurements are made on the same soil in various parts of the electromagnetic spectrum. Complementary to this, in 2010 Sinfield *et al*. [33] published a review on the current state of sensor technology for monitoring nitrate (N), phosphate (P), and potassium (K) status of the soil to optimize fertilizer production inputs. Other specific types of sensors are also able to detect the problem of soil compaction which causes many problems in crop production (e.g., decreased water storage and soil physical fertility). Hemmat and Adamchuk [23] gave a detailed classification of available sensors for measuring soil compaction.

Wireless sensor networks for measuring soil properties had been mainly developed for soil water content and soil temperature [34]. Continuous *in-situ* sensor measurements would be able to provide real-time information on field conditions which can be used as a direct input for farm management systems. For example, Moghaddam *et al*. in 2010 [35] presented a smart wireless sensor web for optimal measurement of surface-to-depth profiles of soil moisture using *in-situ* sensors. The starting point was to develop a sparser but smarter network with an optimized measurement schedule in space and time which can provide soil moisture estimates by operating sensor scheduling and estimation strategies. Ritsema *et al*. in 2009 [36] presented an advanced approach with a communication protocol including data transfer to an end user web page which is applied for continuous monitoring of soil water contents at multiple depths. Finally, Sun *et al*. in 2009 [37] have presented a solar-powered wireless cell that contained a water content sensor, two temperature sensors, an optical sensor, a communication module and a data-logger. Experimental results under varying weather conditions showed that the cell is promising to be used as a node of wireless network in agricultural fields.

Real-time commercial proximal soil sensing systems have already been developed for various operations in crop production. In general these techniques are less accurate than laboratory analyses, but they improve the efficiency of soil data collection and provide the farmer spatial patterns of soil variation instead of only a few accurate measurements. In addition, by combining these soil patterns with observed yield patterns, stratification zones can be identified for spatially varying management [27]. Recent advances in image based sensing techniques showed the potential to map soil properties like soil organic matter from airborne [24,38] or spaceborne platforms [39].

Currently used, off-the-shelf soil moisture sensors, such tensiometers, electrical resistance sensors and dielectric sensor, are also relatively large, need soil-specific calibration and careful installation with good connection with the soil and are often wired making them impractical for agricultural fields [40]. The radio signal attenuation hinder data transfer from networks installed underground. However, wireless data transfer through the soil has developed fast in recent years [41–44]. Ritsema *et al*. [36] and Tiusanen [43] have reported good performance of underground soil sensor networks in their tests. The communication (at ∼868 MHz) through soil showed some problems, especially if distances between underground and aboveground nodes were longer. The tested distances were between 6–62 m and 30–150 m, when sensor depths varied between 4–10 cm or 10–40 cm, correspondingly [9,36,43].

#### 2.1.2. Plant Protection

Control of disease, insect pests, and weeds is an essential operation to gain a stable high yield of crops and high-quality products. To be able to destroy a weed or disease, it has to be first sensed or detected. Different sensing techniques are used to sense the position of plants and differentiate weeds in order to appropriately apply protection agents. These sensors and actuators are either attached to the tractor or are independent autonomous machines for this purpose. Spectral colour analysis techniques [45,46] and textural properties of leaves [47,48] have been employed to identify weeds. Gerhards and Christensen in 2003 [46] and Lamm *et al*. in 2002 [49] used the shapes of plants to differentiate between weeds and crops.

After sensing the unwanted weed or disease, mechanical, chemical or thermal methods can be used to destroy these weeds. Slaughter *et al*. [50] reviewed different methods used for weed control. Rotating hoes and tine types of weed control actuators has been utilised for weed control [51–54]. As reviewed by McCarthy *et al*. [55], commercial weed control systems on the market include Photonic Detection System [56] that uses reflectance in the NIR wavebands to determine the spectral differences between weeds and the bare ground, the Weedseeker [57] which employs light emitting diodes to assess the ratios of red and NIR reflectances of weeds and background, and the Rees Equipment [58] that identifies weeds based on colour, basic shape and size properties using video image analysis. Also a tractor-mounted pendulum-meter which measures plant biomass of crop has been tested for controlling fungicide dosage in variable-rate application [59]. Lamm *et al*. in 2002 [49], Gerhards and Christensen in 2003 [60], Luck *et al*. in 2010 [61] used prototype precision chemical application system with microscopic or boom section control in a test of a robotic weed control systems to improve the precision of agent application. Available thermal methods for weed control use similar sensing but different types of actuators and mechanisms for weed control. Heisel [62] described in his research the use of non contact laser treatments methods for control of weeds.

#### 2.1.3. Fertilising

Considerable progress has been made in the use of proximal remote measurements with handheld and tractor mounted sensors for nutrient management in arable farming [63,64]. For these so-called near-sensing systems, different commercial devices are currently on the market (e.g., Yara N-sensor, Greenseeker, Cropcircle, Isaria) which measure reflectance in a small number of relatively broad spectral bands using their own active light-source. In an operational setting, the sensors are mounted on the tractor or the spraying boom and measurements are acquired when agricultural activities on the field are carried out [65]. This results in a regular point sampling of the field depending on the number of sensors and distance between them and the velocity of the mobile platform during acquisition. The output of near-sensing instruments consists either of more general vegetation indices like normalized difference vegetation index (NDVI) and red-edge position (REP) or system specific indices that represent the relative difference in crop conditions. Based on this relation, variable-rate technology is developed for spraying and fertilization based on real-time sensor data acquisition [24].

Most studies focus on the use of near-sensing systems for optimization of nitrogen fertilization in arable crops like wheat [66,67], corn [65,68], potato [64] and cotton [69] but also for automated fertilizer application in tree crops [70]. For example, in 2006 Berntsen *et al*. [67] adopted the Yara N-sensor to target nitrogen fertilizer in fields of winter wheat. Their results showed that better relationships between sensor measurements and grain yield could be achieved when improved sensors would be able to describe additional crop features (e.g., LAI and canopy characteristics) or soil properties (e.g., soil organic matter, water content). An inter-comparison study by Tremblay *et al*. in 2009 [65] showed that although both commercial sensors (Greenseeker and Yara N-sensor) were capable of characterizing differences in crop growth resulting from variation in nitrogen status, marked differences between sensors were observed in NDVI development over the growing season. The studies indicated that integration of near-sensing instruments or the combined use of near-sensing systems with remote sensing data sources still require further improvement [63]. In addition, recently published vegetation indices like the Normalized Area Over reflectance Curve (NAOC) [71] and the Double-peak Canopy Nitrogen Index (DCNI) [72] combined with biomass indices (e.g., weighted difference vegetation index (WDVI) [73] showed promising results to assess crop nitrogen status. To improve site-specific nitrogen management, plant growth models require accurate information on the whole cropping system, including the crop nitrogen status, and supply and losses from the soil with high temporal and spatial resolution [74].

#### 2.1.4. Irrigation

Efficient irrigation management needs information on when and where to irrigate and how much water to apply. Sensor information on soil characteristics, mostly soil moisture and temperature, can be utilised in deciding timing and amount water applied as well as in validating irrigation efficiency. Also indirect sensor measurement on crop stress has been developed for irrigation practises [40,75].

The potential of sensor technology for irrigated agriculture has been studied since mid 80s [76,77]. Since then rapid development of communication technologies has replaced wired soil conditions monitoring systems with wireless systems. In Spain such an experimental system for monitoring soil, weather and irrigation water was developed for precision horticulture. The system provided more comprehensive data in both space and time compared to the traditional systems based on manual measurement with portable device. The wireless techniques made systems faster to deploy and provide flexibility while challenges were found in costs of the sensors, lack of experienced staff and in power supply [78].

Holler presented [79] a ten-node soil moisture and temperature monitoring network for managing regulated deficit irrigation in Californian vineyards. They used inexpensive off-the-shelf sensors for optimizing irrigation duration and intervals and for monitoring water sources and irrigation system operation (e.g., line pressure, tank filling). The use of sensor data seemed beneficial as the sensor network was extended to two adjacent vineyards. Benefits were gained from improved grape quality and yield and from savings in water use, pumping energy, and labour and repair costs.

In conventional irrigation, fields are managed uniformly both in terms of water application depth and timing, which may results in over- or under-irrigation. Site-specific irrigation management takes into account spatial variation by using management zones that are treated individually [80]. Vellidis *et al*. [81] presented an irrigation scheduling application based on soil moisture and temperature sensors. Sensor data was used to define irrigation zones, timing of irrigation and amount of water to apply. The systems used RFID technology in transmitting monitoring data which had an advantage of decreasing cost and power use by the transmitting network. They also developed variable rate irrigation (VRI) system retrofit package that is suitable for most existing pivots to allow varying water application amounts. The VRI system was also commercialised in United States [81,82].

Control of irrigation system on basis of sensor measurements requires wireless real-time communication. Automated irrigation system should also be able to allow a variety of control inputs (also sensor inputs), to control individual nozzles and be aware of its location to optimise amount of water applied [81,83,84]. Decision support software for the control of linear-move irrigation based on near real-time sensor networks was described by Kim and Evans in 2009 [84]. The experimental system employed wireless sensor network of soil moisture monitoring, and used sensor data to adjust irrigation and nozzle sequencing for performing site-specific irrigation. The location of irrigation machine was also monitored and the system was adaptable for different crop designs, irrigation patterns and field locations. Chavez *et al*. [83,85] recently described an automated control system for lateral-move irrigation systems. It controlled on/off time of each nozzle to create spatially variable water application rates. The system worked at 1-min resolution and integrated *in-situ* soil moisture sensor data, remote sensing maps with thermal infrared readings, spatial information on field characteristics and static information of crop water use to automatically provide a map for controlling irrigation task and providing feedback on tasks’ success.

Irrigation management posed requirements for sensor and communication technology: sensors should be easy to use and install, expenses of sensors be low, power-use of instruments and communication be low and data representativeness and reliability be at reasonable level. Interoperability of sensors and sensor networks with irrigation control systems also need development [84]. The benefits of sensor measurements were obtained by increased efficiency of water use [83]. There is also evidence that site-specific irrigation can increase amount and quality of yields for crops that are sensitive to soil water availability such as potato. However, increase in income may not cover the costs of monitoring and improved irrigation systems [80]. Site-specific irrigation had also lowered risks of plant diseases and environmental damages, such as nutrient leaching and soil compaction [40,80,84]. For site-specific irrigation, as any precision agriculture technique, correct modelling of spatial variability (of soil moisture) is critical, otherwise uniform application rate may be more approved than variable rate applications [75].

Remote sensing was also studied in mapping crop water status. Thermal imagery provides spatial data on surface temperature which was used to estimate crop water status. The review of recent remote sensing studies on crop water status is given by Lee *et al*. in 2010 [14].

#### 2.1.5. Harvesting

Yield sensing and mapping during grain harvesting has been extensively studied and is also widely adopted practise. Yield monitor systems usually incorporate data from a yield sensor, a moisture sensor, a ground speed sensor, a cut width sensor, an elevator speed sensor, and a differential global positioning system (DGPS) receiver to relate sensed grain yield to location [86]. Various techniques for on-the-go yield sensing were described in review articles by Arslan *et al.* [86] and Reyns [87]. Reyns [87] separated mountable grain yield sensors in four groups: Mass flow measurements, volume flow measurements, impact sensors and indirect methods, where indirect methods includes radiometric and capacitive methods. Reyns [87] gave a detailed overview of published research on different sensors as well as an overview of commercial sensors on the market. Most major agricultural equipment companies now provide optional yield-mapping systems for their combine harvesters. A fair amount of studies have been conducted to assess the accuracy of yield measurement and yield mapping (see [86,88,89] and references therein).

In addition to crop yield, also sensing of crop quality has been developed. For grain crops, these applications typically give information on crop moisture and protein content. In commercial applications, grain protein content monitoring is carried out with sensors based on either Near-Infrared Transmittance (NIT) on stationary grain samples (Cropscan 2000H and Zeltex Accuharvest) or NIR technology measuring from continuous flow of harvested grain (The ProSpectra) [90]. Taylor *et al*. [91] tested on-harvester grain protein and moisture sensing using NIT technology (Zeltex AccuHarvest). They reported coherent output and strong indication that observed patterns are real, and pointed out that local calibration is needed for the sensor. Whelan *et al*. [90] studied use of the same harvester-mounted on-the-go sensor to assess wheat grain protein concentration, grain yield and their site-specific variation. With calibration to local (Australian) crops, they managed to get consistent estimation of protein level with measurement precision better than 0.5%, over a large grain protein content range. Similar levels of accuracy were reported by Long *et al*. [92] as they were using NIR reflectance spectrometer (ProSpectra Grain Analyzer) to measure wheat protein content in a moving grain stream.

On-the-go measurement of grain quality in harvesters could also allow separation of crop into quality classes, and thus bring competitive advantage if there are non-linearity’s in the payments of quality premium [91,93]. Measuring quality may also help explain the variation in crop yield [87], enable nitrogen fertilizer management, and support environmental auditing at the within-field scale [89]. Crop quality sensing was regarded as one of the future trends in precision farming [93–95]. Research on sensing and yield mapping applications for forage crop harvesting has been lesser in amount. Some recent examples are [96] and [97]. Lee *et al*. [97,98] developed a wagon-based real-time yield monitoring system that was successfully implemented in a silage corn field. The system used moisture sensors, DGPS receiver, load cells, and Bluetooth modules for wireless data transmission. In addition to yield and crop quality monitoring, emphasis of the research is on how to interprete yield maps, and convert them into actionable data such as management plans [99,100].

While harvesting of grain and forage crops utilized on-the-go sensors, in viticulture sensor network applications were emerging. However, full-scale decision support systems (DSS) that could convert sensor monitoring data into decision support on harvest still remains a challenge, also in viticulture [101].

Precision viticulture has to deal with the existence of variability of grape yield and quality, often with more financial importance on quality. Matese *et al*. [102] presented a sensor network solution for precision viticulture that was set up on four experimental vineyards in Tuscany, Italy. The system included a base agro-meteorological station as a Master Unit, and a series (10) of peripheral wireless nodes (slave units) located in the vineyard that monitored air, grape, leaf, and soil temperatures, soil water potential, grape radiation, leaf wetness and wind speed. The Master Unit utilized wireless technology for data communication and transmission with the slave units and remote central server. The system was originally developed to monitor agro-meteorological parameters and environmental conditions in different management practices, to see how they influence grape quality. The network was seen to be flexible and customizable, and it was also possible to set an alarm threshold for agro-meteorological conditions such as frost risk [10] or drought [81], or weather conditions leading to increased pathogen risks [103] and thus provide additional, actionable data to the farmer.

Burrell *et al*. [104] explored usability of sensor networks in terms of user needs, equipment capabilities, and environmental conditions for a vineyard in Oregon, USA. In the study they considered several optional system architectures, and carried out a trial installation of 18 monitoring motes capable of in-network processing of data, and using radio frequencies for data transmission. It was concluded that even if sensor networks provide valuable information on the optimal timing of harvest, the decision of actual harvest time should not be fully automated, mainly because it is often a subjective and social-economically-based decision.

#### 2.1.6. Fleet Management and Control

Fleet management and control Systems (FMS) encompasses the management of a company’s mobile vehicles properties, which includes the purchase, maintenance, inventory, disposal and work scheduling of these vehicles. Fleet Management, which became widely adapted in industrial domains from the end of the 1980s, provides added value for an enterprise or organization by improving efficiency and productivity of mobile vehicles [105,106]. Just as has happened in the transportation industry, agricultural production has undergone significant changes to embrace large farms with multiple machinery and chains that require more efficient management. Furthermore, modern agriculture machinery is advanced and equipped with various smart sensors some of which are wirelessly networked [107].

Fleet management in agricultural crop production consists of two main components a transport telematics; located on the vehicle, which serves and receives network information, and a software application [108]. The software application can be used via local wireless networks or through the internet to provide access to the fleet to be managed [109]. The transport telematics consists of a positioning system; a key component for fleet management, and a series of networked sensor nodes that enable process and environment monitoring [18].

Large and intensive crop production farms invest heavily in machinery; therefore, their usage and maintenance must be efficiently planned and implemented. For efficient management of the fleet, enterprises need information about where their mobile vehicles are, how they are operating, and in which environment they are operating. As the agricultural machinery has become complex and diverse, the International Standards Organization has charged the Technical Committee 23 (ISO/TC23/SC19/WG5) with standardizing wireless communication amongst agricultural equipments. Fleet management and implement synchronizations are one of the priorities in the standardization work.

Within this scope, fleet management with WSN in agricultural crop applications a can be classified into two main groups: firstly plant production vehicles and equipment, and secondly unmanned aerial vehicles (UAV). Plant production vehicles and equipment perform field operations such as soil manipulation, planting, fertilizer and disease control applications, and crop harvesting. UAV’s monitor crops and environmental conditions of the crop production system.

For plant production vehicles and equipment, recent publications have reported the trends in the management and control of fleet in farming operation which involve single or multi-machinery systems of combine harvesters, self-propelled, tractor-trailer units and one or more crop transport units. Guo and Zhang [110] presented in 2005 a wireless data fusion system that automatically collected and processed operational data from agricultural machinery in order to provide real-time support for precision farming tasks. The system provided transparent data for management and decision support functions to crop producers by wirelessly integrating a machinery-based sensing unit and an office-based data processing unit. The machinery-based unit collects the machine position and altitude data, the office-based unit performs data fusion to accurately estimate the machine position, and the wireless data-link transmits the data between the machinery- and the office-based units. Field tests indicate that this system can collect field data from the operating machinery and wirelessly send the raw data to a data-processing centre to acquire accurate machinery positioning data at a sampling rate of 50 Hz.

Apart from research literature and prototyped trials, major agricultural machinery companies are becoming key actors in fleet management in agriculture. Examples include Claas group’s “Agro-Combine online” and the “Claas Telematics” [111] or John Deere’s “JDLink”.

For unmanned aerial vehicles, Techy *et al*. [112] described the use of a control strategy (coordination via speed modulation) to synchronize two autonomous fleet UAVs during aerobiological sampling of the potato late blight pathogen, *Phytophthora Infestans*. The UAVs shared position coordinates via a wireless mesh network and modulated their speeds so that they were properly phased within their sampling orbits. Their report was one of the first using an autonomous UAV coordination for aerobiological sampling of a plant pathogen in the lower atmosphere. Göktogán *et al*. [113] also presented a novel application of an autonomous Rotary-Wing Unmanned Air Vehicle (RUAV) as a cost-effective tool for the surveillance and management of aquatic weeds. The presented system locates and identifies weeds on inaccessible locations. The RUAV was equipped with low-cost sensor suites and various weed detection algorithms. In order to provide the weed control spraying and treatment of the aquatic weeds the RUAV is also fitted with a spray mechanism. The system has been demonstrated over inaccessible weed infested aquatic habitats. Hunt *et al*. [114] also remotely controlled and monitored a small Unmanned Aerial Vehicles (UAVs) by acquiring digital color-infrared photographs from a single 12-megapixel camera without an internal hot-mirror filter but with a red-light-blocking filter in front of the lens, resulting in NIR, green and blue images. The UAV-camera system was tested over two variably-fertilized fields of winter wheat and the authors found a good correlation between leaf area index and the green normalized difference vegetation index.

In their research Huang *et al*. [115] presented an overview on the development of three UAVs for crop production management. The remote monitoring of the UAV’s were also discussed. They presented an ultra low volume sprayer for a UAV helicopter. Two other UAVs, one helicopter and one fix-wing airplane, were evaluated for low-altitude remote sensing over crop fields. The integration of the spray technology and the remote sensing technology on the UAV systems provided a great potential to identify crop stresses and hence spray crop production and protection materials at different rates over small crop fields to realize highly accurate site-specific crop production management. Lelong *et al*. [116] focused on the use of a combination of simple digital photographic cameras with spectral filters, designed to provide multispectral images in the VIS-NIR domains in light-weight UAV for remote sensing for precision farming. Successful tests were performed on ten varieties of wheat, grown in trial micro-plots in Southwest France.

### 2.2. Use Cases

We present two cases of using sensor measurements in agricultural applications in detail and conclude with the lessons learnt during sensor network deployment and maintenance as well as application development.

#### 2.2.1. Precision Farming of Potatoes in The Netherlands

In crop production, nitrogen is most frequently the limiting nutrient and is needed by most crops at higher quantities than other nutrients. Optimal nitrogen supply is essential to secure abundant yield with high quality. For example for potato, which has a relatively low nitrogen use efficiency (50–60%) due to its naturally shallow and poorly developed root system, insufficient nitrogen exhibits yield while excessive nitrogen applications can reduce both yield quantity and quality [64]. Over the last decade, significant progress has been made in sensing methods to detect and diagnose crop nitrogen status for precision agriculture applications. This refers to remote sensing based methods from spaceborne or airborne platforms which are the basis for operational services like Mijnakker in the Netherlands (www.mijnakker.nl) and GEOSYS in France (www.geosys.com), but also includes the use of handheld and tractor mounted near-sensing (or ground-based) sensors for which a range of commercial devices (e.g., Yara N-sensor, Greenseeker, Cropcircle, Isaria) are currently available on the market.

To investigate the application of near sensing instruments for fertility management in potato, a field study was conducted on an arable farm in the south of The Netherlands. The objective of the study was to investigate to which extent tractor mounted near-sensing instruments like Greenseeker (GS) are able to detect and monitor differences in fertility management practices in a potato crop. In addition, the complementary use of optical sensors and data from sensor networks to diagnose potato crop status over the growing season was evaluated. The GS sensor measures crop reflectance using an integrated LED emitting light in the red (656 nm) and NIR (774 nm) band from which the well-known NDVI can be calculated. Six GS sensors were mounted on the spraying beam behind the tractor resulting in a regular point sampling of the field depending on the velocity during acquisition. Sensor measurements were acquired during regular agricultural management activities (e.g., fungicide application) resulting in a frequency of 11 measurements over the growing season of 2009. In addition, continuous meteorological data (e.g., temperature, rainfall) were available from an on-farm sensor network which was also used as input for a decision support system to optimize use and timing of fungicide applications to mitigate the risk of potato late blight disease development.

**Figure 1.**
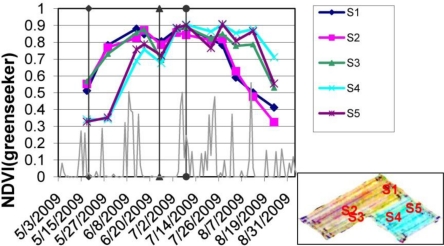
Development of normalized difference vegetation index (NDVI) from time-series of measurements with Greenseeker sensor in potato parcel and comparison with rainfall data from nearby meteorological sensor network. The timing of agricultural management practices (e.g., irrigation and fertilization) is also indicated in the figure. The inset shows the sample locations for the Greenseeker sensor within the parcel. The variation in colours represent a composite image indicating NDVI at different stages over the growing season: red: 13 June; green: 9 July; blue: 29 July 2009.

A simple experiment was set-up to track potato crop development over the growing season and to evaluate effects of weather on crop development. One parcel was split in two parts and planting dates were varied with early planting in the western part (April 25, 2009) and later in the eastern part (May 13, 2009). In addition, soil conditions (e.g., organic matter, moisture) within the parcel were spatially varying. Figure 1 shows the development of NDVI as an indicator for vegetation biomass measured with the GS sensor over the growing season for five spatially varying locations within the parcel. The composite image in the inset of Figure 1 which combines the spatial distribution of three NDVI observations over the growing season clearly detects the spatial variation in potato crop status caused by the difference in planting date and the inherent soil variability.

Additionally to the spatial variation, also the temporal development of the crop varied significantly at different positions within the field (Figure 1). A clear difference can be observed between S1, S2 and S3 (early planting) and S4 and S5 (late planting) in NDVI development where the first samples reach a NDVI saturation level of 0.85 around mid June and the latter mid July. However, for all locations a clear reduction of NDVI can be observed between mid and end of June. Comparison with rainfall data from the meteorological sensor network shows that this overlaps with a period with no rainfall. Based on this information, the farmer decided to apply irrigation for the whole parcel which resulted in a quick recover of the crop as shown by the increasing NDVI. Although some effect of nitrogen fertilization can be observed after the application of mid July especially for S4 and S5, the detection and diagnosis of nitrogen status of the potato crop needs further improvement. Firstly, by the use of optical vegetation indices (e.g., red-edge position) which use spectral bands related to the crop nitrogen status, and secondly, by analyzing detailed multi-temporal sensor measurements [74]. The latter would require the integration of remote sensing and (mobile) near-sensing measurements to achieve the best possible consistent high-frequency time-series for an improved continuous detection of crop status.

The results of this case-study indicated that complementary application of optical sensors and sensor network is promising to optimize management activities and the usage of (natural) resources in arable farming. As the modern farmer requires near-real-time and ready-to-use information to drive management decisions and it is anticipated that sensor costs will decrease considerably over the coming decade, the next critical step is to improve the quality of sensor measurements in precision agriculture. Therefore, based on the presented case study and scientific literature, the following aspects would need development:

Due to different reasons the timeliness of sensor data is still limiting: e.g., remote sensing is weather and satellite dependent and near-sensing is dependent on management operations;

Currently available sensors and related services are mostly based on one or a limited number of sensor data streams while a combination of sensors will be required to detect and diagnose processes within the soil-cropping system. This will require innovative data analysis methods to take advantage of complementary sensor data streams;

Established relations between sensor measurements and crop-growth, -composition and -yield need improvement. For instance by combining existing databases and developing more flexible diagnosis methods which are fitted to management information needs of the individual farmer;

The availability of directly useable spraying maps and information for site-specific management derived from sensor measurements needs improvement. Therefore, new concepts of information organization are required which take advantage of the increasing opportunities for web-based data processing (e.g., cloud computing) and wireless communication between sensors and devices.

#### 2.2.2. Applications of the SoilWeather Sensor Network in Finland

**Figure 2.**
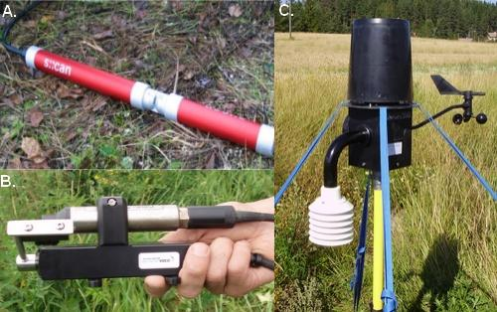
Photographs of the devices used in SoilWeather WSN: (**A**) the spectrometer used in measuring water turbidity and nitrate concentration; (**B**) Turbidity sensor; and (**C**) weather station (Photographs: Lippo Sundberg, MTT Agrifood Research Finland).

Testing and development of agricultural forecast models and automating the use of sensor data in agricultural applications calls for reliable sensor measurements that can capture both temporal and spatial variation in the area of interest. Several application pilots were carried out using Finnish, large-scale (2,000 km^2^) wireless sensor network, called SoilWeather. The network was established in Southern Finland during the years 2007 and 2008 to support development of environmental monitoring, agricultural forecast services and precision farming, and also to provide local farmers timely information on local weather parameters. SoilWeather wireless sensor network (WSN) monitors local weather parameters (air temperature, humidity, precipitation, wind speed and direction), soil moisture and parameters for ditch and river water (turbidity and nitrate concentration) at a frequency of 15–60 min (Table 1, Figure 2). The nodes of the network (∼70) are mostly compact weather stations (a-Weather) that are located in the fields of private farms. GSM network is used in transferring data from nodes to database. Data is available near-real time (time lag 1 h in maximum) at customized web services. Every 24 h, the data is validated by simple data quality algorithms flagging missing data and erroneous data (such as measurements outside of defined ranges). Maintenance of SoilWeather WSN measuring stations is carried out at a regular basis (twice a year) and according to the alerts from quality control system. Common maintenance tasks are replacement of batteries or broken parts and manual cleaning of the sensor probes. Especially the aquatic sensors require frequent manual cleaning due to contamination during the growing season, even though the sensors are equipped with automatic cleaning systems based either on a mechanical wiper or air pressure. SoilWeather WSN is described in detail by Kotamäki *et al*. [117].

SoilWeather WSN serves as platform for research and application development including agricultural application for farmers, nutrient leaching studies from agricultural land to water system and retention efficiency studies of constructed wetland [117]. Here we focus agricultural applications that can improve crop production processes at farm level, and thus emphasis is on weather and soil measurements instead of water measurements.

To demonstrate the usability of local sensor measurement in agricultural decision support, we carried out a plant protection forecast pilot for potato blight, white mould of turnip seeds and three leaf blotch diseases (glume blotch and tanspot for wheat and net blotch for barley). In potato blight application, timing of the spraying operation was supported by estimates for onset and spreading of potato blight We employed the complex LB2004 model at Web-blight warning service (www.euroblight.net) [118,119] in which an interface was built to operate with SoilWeather sensor data (air temperature and humidity). Additionally, potato blight risk was assessed by a simple model that uses only temperature and relative humidity measured locally in farms. This model was tested for 11 potato fields in SoilWeather WSN. The prevention need for white mould of turnip seeds was estimated by a simple decision rule: if the soil is continuously moist (precipitation is ∼30 mm) in three week time period before the flowering of turnip seed, prevention against white mould is needed [117].

The crop disease prognosis for wheat and barley provides a daily estimate for accumulated risk for infection. Prognosis is based on the weather data from SoilWeather WSN (temperature, humidity and rainfall) and cultivation data from FMIS on sowing, cultivation history, tillage and cultivar. Web-based FMIS can access SoilWeather WSN database. The prognosis model was verified by observations made in 18 farms. The farmer was alerted when risk level exceeded the predetermined limit. For cases where a farm does not have an own local weather station, spatial coverage was arranged by using national weather data. Plans to productize the application as a part of a commercial WebWisu FMIS exist.

A grass harvesting time model and service application (Artturi, https://portal.mtt.fi/portal/page/portal/Artturi/artturi_web_service) is operational with national weather data and available for farmers. The empirical model estimates concentration of digestible organic matter of forage based on effective temperature sum. In the context of SoilWeather WSN, research on this application is focused on the possible additional value gained from higher spatial or temporal frequency from the sensor data.

Automated use of local weather parameters was also piloted in agricultural on-field operations. In the pilot, local weather information was used as decision support for farmer to optimize pesticide spraying task by taking into account wind drift of the agent and safe time for spraying. A prototype task controller for executing precision spraying tasks was developed and data transfer between SoilWeather database, FMIS and a field vehicle was demonstrated. In addition to other relevant information such as sprayer information and GPS location, the task controller of a work set provides the farmer information on weather parameters from local weather station of SoilWeather, and rainfall forecast for next three hours.

The experiences gained from building up the sensor network and during four years of operation has highlighted the value of sensor data, but also revealed that resources have to be allocated for the maintenance of sensors and data quality control. The added value was gained more on temporal and spatial resolutions, which made it possible to rapidly analyse or observe highly variable phenomena, such as nutrient leaching peaks, and integrate local measurement data (e.g., farm weather station) to agricultural models and with work-sets.

In SoilWeather WSN, running costs include repair of the devices, communication network costs (SMS messages), data service cost, and work time and travel costs used for maintenance and cleaning. Even if actual costs of sensor devices are decreasing, it should be emphasized that greater part of overall costs accumulates from maintenance of network, thereby creating the need for long-term financing.

The SoilWeather WSN produces about 30,000 measurements daily. With this rate of incoming data, the data quality control need to be automated as far as possible, but still some expert knowledge and manual measurements are needed to keep track on the need for maintenance and overall quality of the measurements. Variation in data quality needs also to be taken into account in the development of automated applications. Software applications have to deal with temporal gaps in the data stream e.g., due to communication network breakdowns, and with spatially irregular gaps as sensor networks are still relatively rare. In the developed plant protection forecasts, national weather data was used when no local sensor data was available. This highlights the need for interoperability between data sources and standardised interfaces enabling integration of different data sources for applications. Sensors also provide erroneous measurements e.g., due to the contamination of sensors, of which most should be identified by data quality algorithms. Although data quality algorithms need to be developed further, there is also a challenge of communicating data quality to the end users.

## 3. User Requirements

Even if research on sensors and sensor networks in agriculture covers a wide spectrum of applications, they only have real potential to become operational applications when they provide economical or other benefits for users, such as decreased work load for farmer or environmental effects of crop production. In crop production, added-value from sensors and sensor networks was expected to be gained by increased efficiency in use of water, agrochemicals, nutrients and farm vehicles and by improved yield both in quantity and quality resulting to benefits both for farmer and environment. All the sensor types, *in-situ* sensors, near-sensing and remote sensing were employed, and several commercial sensor products were available. Sensor technology was used more in specialty crop production, such as vegetables, fruits and grapes, than in field production. The reason is that in specialized crop production, added value is higher, which makes investments in sensor technology economically beneficial. Based on the literature review and our own experience from the use cases we have identified eight user requirements for successful deployment of sensor technology especially of sensor networks.

### 3.1. Ease of Deployment and Maintenance of Sensors

Easy installation and use of sensors was considered an important issue for operational and wide-spread applications. Whereas sensors and networks were considered to be flexible and suitable for many purposes and locations, technical expertise needed in deployment and use was seen as drawback. At the moment plug-and-play sensors are only limitedly available. In traditional farming, many tasks in crop production processes can be carried out by unskilled labour. Automation and use of sensors may decrease these job vacancies while creating demands for higher-skilled and more specialised workers [14]. The lack of technical expertise and support for farms was often raised as one of the major challenges in taking sensor technology into operational use in farms.

Several technical solutions such as solar energy harvesting, automatic cleaning systems, development in materials (e.g., antifouling materials), automatic calibration and remote control of sensors has been developed to decrease maintenance efforts, yet still all the sensors need some amount of maintenance. Deployment and maintenance may also be outsourced so that installation and maintenance comes along with the measurement service. However an arranged maintenance causes costs and requires resources over the measuring time-span. In some cases, field work may increase compared to the traditional sample based analysis [2,117,120], but on the other hand the obtained level of information is higher.

Power supply was commonly seen as a main bottleneck in literature, and thus, solutions was searched to either decrease sensor energy use or increase their energy use efficiency by improving battery technology or by solar harvesting systems [37]. In addition, energy use of sensor nodes was commonly improved by limiting communication distances or measuring frequencies and by activating the sleep mode of sensors when no measuring needs exists.

### 3.2. Sensor and Network Reliability

Sensor nodes need to be durable and rugged for varying weather conditions, in some cases under dirty and vibrating field vehicle conditions, and against animal disturbance and destruction. This demands for a good design and casings for sensor nodes, but also allowance for maintenance and cleaning of sensor devices. Sensors work reliably only if they are correctly maintained.

Sensor networks were mostly wireless systems with real-time or near real-time data transfer via radio, Wi-fi, ZigBee, Bluetooth or 3G mobile phone networks. The optimal data transfer solution needs to be considered case by case considering issues like power consumption, network topology, number of nodes, sensor and data type, data transmittance distances and need for extenditibility. The benefits and challenges of these technologies has been widely discussed in existing reviews [17,18]. Additionally RFID based communication was tested in an irrigation application [81]. Wireless communication made devices easier to install and less demanding for measuring sites. It also decreased costs because wires were not needed. However, coverage of wireless networks is still low in many rural areas [14]. Reliability of communication has often been questioned, but in the application review it did not appear to be a major bottleneck (keeping in mind that most applications were short-term experiments), although some problematic experiences have been reported [121]. Many reviewed cases were prepared for packet loss or failures in communication connection e.g., by employing diagnostic software for observing problems or alternative routing possibilities for sending measurements.

### 3.3. Data Correctness

Data quality can be considered as one of the most critical challenges related to sensors applications and networks [122], and especially when data is used in decision making or, moreover in automated processing e.g., on-the-go variable rate application. Sensors may produce erroneous measurements or there may be gaps in data streams, e.g., due to sensor, software or communication network failure. Due to changing natural conditions such as high dynamics of ambient light, field-based sensors will not provide accuracy similar to conventional, laboratory based methods, but may provide spatial and temporal coverage and timelines that cannot be obtained by conventional methods e.g., on soil properties [23]. The spatial accuracy of sensor data as well as GPS location field vehicle is critical for site-specific applications: in case of spatial inaccuracy, tendencies are that it is cost-efficient to apply equal levels of nutrients or chemicals [75].

Data quality is a result of several aspects: selection of appropriate measuring sites and sensors, proper deployment, careful sensor calibration and maintenance, and reliable data transfer and management. Some of the challenges are purely technological, e.g., data transfer, whereas others are more related resources, skills or ways of action. Limited data quality due to loss of calibration accuracy could be solved by automated calibration of sensors [123]. However, focus should also put on selection of appropriate sensors and careful deployment in representative sites considering parameters measured.

Sensor observations vary in quality, which has to be taken into account in data delivery e.g., by specifying quality flags for observations. Although data quality algorithms to flag erroneous (or missing) observations were well-developed in meteorological networks, these DQ tools were less frequently applied to sensor networks in other domains. However, for end-users, being a farmer, a model or a machine, it is critical that data quality is communicated.

### 3.4. Data Amount

Continuous data acquisition in sensor networks results in fast accumulating amounts of data which poses a challenge to data management. One of the solutions to limit data amount was *in-situ* processing or filtering of data, in particular when data sets were very large or data is acquired with high frequency. In the cases of on-the-go sensing and applications from mobile vehicles like tractors, *in-situ* processing were a requirement. However, this will result in an increase of energy consumption which often is not problematic for moving vehicles but could be a limitation for wireless sensor networks.

Data amount was also limited by timing measurements to events. Thus, most sensor nodes of the network were turned off until something interesting was observed. Sensor data availability is also supposed to increase due to changes in data policies and in ways of collecting and sharing data. Larger amount of data available recalls tools for finding data, and for data analysis e.g., by data mining and fusion.

### 3.5. Interoperability and Standardization

The availability of equipment with more computing power makes it possible to utilize various sensing techniques such as global positioning systems (GPS), machine vision, weather sensors, dead-reckoning sensors, laser-based sensors, inertial sensors and geomagnetic direction sensors for applications in agriculture. Heterogeneity of sensors poses also many challenges for data integration and interoperability of systems: data of wide spectrum of variables is generated in various formats and resolutions by sensors using different measuring technologies and standards. Moreover, data is transferred between various hardware, software and organizations, such as FMIS, farm machinery and vehicles, and external web services, resulting in incompatibilities [124]. In order to widely apply sensor technologies in agriculture, there is the need for standardizing sensor communication and data produced.

Interoperability, data transfer and data harmonization are the major problems to be addressed for enabling easy data transfer between sensors, agricultural machines and the agricultural supply chain network, although some progress had been made in recent years. For crop production machines, the ISOBUS standard adresseses data exchange and compatibility between hardware systems for crop production in agriculture. ISO standard ISO 11783 specifies the data BUS communication standard between the tractor and implementation chain. However, the integration of different networks from external intelligent systems such as environmental sensors amongst others is still not widely available [125]. European Union’s (EU) INSPIRE directive aims to harmonize European spatial data infrastructure. This will enable the sharing of environmental spatial information among public sector organisations and better facilitate public access to spatial information across Europe [126]. Geography Markup Language (GML) has also been proposed to serve as a good basis for standardising data formats for precision agriculture [18]. OGC’s standardized web services and interfaces are commonly recognised and the following have high relevance in agriculture: WMS (Web map service), WFS[-T] (Web feature service [transactional]), WCS (Web coverage service), WPS (Web processing service), GML (Geographic markup language), ISO 19115 (Metadata standard for geographic data sets), SFS (Simple features specification) and CTS (Coordinate transformation service) [127]. Steinberger *et al*. [128] and Ruiz-Garcia *et al*. [18] demonstrated this with the acquisition of agricultural process data using ISO 11783 equipped tractor in ISOBUS XML form, transfering the data to a Farm Management Information System, converting the data to AgroXML form with GPS and open web services based on OGC standards.

### 3.6. Temporal and Spatial Resolution

High spatial and temporal resolution is a well recognized advantage of *in-situ* sensor data. Development of applications was clearly focused on applications that are time-critical and benefit from high measurement frequency or real-time data transfer like alerting services for irrigation or plant protection. High measuring and data transfer frequency enabled to react to rapid or unexpected events, to improve timing of practices e.g., initiate frost prevention and automated adjustment of field vehicles according to the changing conditions.

When considering *in-situ* sensor networks, high spatial resolution was mainly obtained for small-area applications. For example, few parcels may be highly instrumented for analysing soil moisture. Thus, coverage of high-frequency *in-situ* sensor networks was relatively low. A better spatial resolution and coverage was obtained by airborne or tractor based sensing. The spatial accuracy of remote sensing data as well as GPS location of field vehicles was critical for site-specific applications. Collection of location information in constant intervals from mobile vehicles can cause difficulties. Optimal spatial and temporal resolution and coverage of sensing are in relation to spatial variation of measured parameter, scale of application and sensing method. However, they also influence sensor power consumption, communication solutions selected and costs of measuring.

### 3.7. Costs of Sensing

Costs of sensor networks were reported often, but only for devices, not for operation. Sensing costs consists of investment to equipments, data transfer, network maintenance (including cleaning, spare parts, calibration) and possibly data services. The costs of sensors are decreasing when use of nodes increases and mass production is realized. Technological development is also believed to decrease maintenance need and costs. At the moment, it was considered to be an obstacle that limits the use of sensor technology for small area applications or in testbeds.

### 3.8. Access to Data and Applications

Most of the reviewed applications were made available by Internet giving the flexibility to monitor and manage farm activities, and connect to external services or data sources, such as weather forecasts, plant protection prognosis and to control their own sensors. Moreover, up-to-date information is actively offered to the farmers, e.g., in the forms of alerts. The possibilities of standardized web services were commonly recognized, and because sensors mostly produce spatial data, open geospatial web services were considered especially interesting. Integration of agricultural sensor networks to SWE is still untested, but interesting data such as aerial photos has been made available through WMS interfaces [124].

Access to data can also be considered from the viewpoint of the customers, food chain and authorities. Automatic measuring and web services enable to acquire and share information on how food is produced. The proof on responsibility of food production and traceability of food products may also offer economical benefits to the farmer, if customers or food industry are willing to pay more on products or raw material which is produced in a responsible way. Farm management data is sensitive, and farmers have to be confident that it can only be accessed by defined stakeholders

## 4. Conclusions

At the start of this paper, we formulated four research questions about sensors and sensor networks in the context of crop production: How do they support crop production? What added-value do they bring to production process? How users benefit? Which are the risks and bottlenecks involved?

The review showed that lot of potential is seen in sensors and sensor networks along the crop production chain ranging from soil preparation to the harvesting and from monitoring of growing conditions to automated control of machinery. Also some commercial sensor based products (e.g., near sensing systems) and services (e.g., plant protection services, frost alert services) were currently available. Presently, sensor technology was mostly adopted to support decision making and assist farmer in timing of production practises, such as irrigation or plant protection or in allocating chemicals or nutrients according to sensor observed needs (e.g., site-specific management). Other sensors collected information that can be used in the evaluation of production success (e.g., quality and amount of yield). The most advanced sensor systems were used for automated control or adjust of machines or vehicles. Benefits were then gained through better decisions and by developing production process. Overall benefits were obtained in terms of higher yields, improved quality of yields, decreased input costs and production risks or less work time or load. The sensor based applications may also help to reduce environmental effects or food safety risks by lowering use of nutrients, chemicals, energy and water.

Quality of sensor data and how it is communicated to the end-users has a high importance. Errors in the data can trigger to wrong decisions leading to the inefficient resources use, and increased costs and environment risks. Therefore, in automated systems with low level of human intervention, this must be especially considered. There is a risk that users abandon new techniques because it does not respond to their demands on data or application quality. Operational sensor networks call for well-developed error diagnostics that alert the user on fault situations and maintenance actions needed. Alternatively, commercial measurement services may take care of the maintenance and users’ attention may not be needed.

Another point we would like to raise is the integration of sensing data in applications and control systems used in the farms. It should be easy to integrate local measurement into the application but also the measurement or model result produced by these applications should be easy to integrate back to the farming information or control systems through open interfaces and standard data formats. Standards and interoperability are of the essence that sensor based application are utilised in developing and planning crop production in practise. This is an issue that has been widely acknowledged, and a lot of progress has been made in recent years.

Thirdly, all sensors (both mechanical and electronic) need maintenance and recalibration in order to control the quality of measurements. Maintenance intensity varies a lot and often causes great part of measuring costs. The importance of maintenance was not raised in the reviewed applications, maybe because they were mostly research oriented and applied in short term testbeds.

The review showed a wide spectrum of applications and sensor technologies that have potential to benefit crop production. Although some commercial and operational applications exist, majority of the reviewed applications are still running in the research domain. Further testing on usability, reliability and compatibility of applications in practical domain will finally show when or if the benefits gained from sensor technology overcome costs, maintenance efforts and risks related technology.

## Figures and Tables

**Table 1.** Variables measured and sensors used in SoilWeather WSN.

**Measurement stations and measured variables**	**Instrument used (Model)**	**Number of instruments**
Weather station (a-Weather)		
Air temperature (°C)	Pt100	52
Air relative humidity (%)	AST2 Vaisala HMP50	52
Precipitation (mm)	Davis rain collector	52
Wind direction (Deq.)	Davis Anemometer	52
Wind speed (m/s)	Davis Anemometer	52

Water turbidity station (a-Water)		
Water turbidity (NTU)	OBS3+	16
Water level (cm)	Keller, 0.25 bar	7

Nutrient measurement stations		
Water nitrate concentration (mg/L)	S::can Nitro:lyser	4
Water turbidity (FTU)	S::can Nitro:lyser	4
Water level (cm)	Keller PR36	4
Water temperature (°C)	Luode Consulting Ltd (own product)	4
